# Despair is Not Hopelessness: Autonomy and Behavioral Interventions for Deaths of Despair

**DOI:** 10.1093/phe/phag025

**Published:** 2026-09-10

**Authors:** Alejandro Hortal, Jack M C Kwong

**Affiliations:** Department of Philosophy, Wake Forest University, Winston-Salem, NC, USA; University of North Carolina at Greensboro, Greensboro, NC, USA; Appalachian State University, Boone, NC, USA

## Abstract

The term ‘deaths of despair’ refers to suicide, drug overdoses, and alcohol-related mortality in the context of concern about rising mortality rates. This paper employs a philosophical distinction between despair and hopelessness, and their positive counterparts, hope and hopefulness, to examine how behavioral public policy can offer tools to reduce these deaths and what role autonomy plays in such contexts. The paper introduces a further distinction within hopelessness (desiderative and doxastic) each affecting autonomy in different ways, depending on whether desires, beliefs or both are compromised. The analysis considers autonomy from two perspectives: first, how it is diminished in the psychological and social contexts that give rise to deaths of despair; and second, how it may be further compromised by behavioral interventions that rely on paternalistic influence. By integrating these conceptual and ethical dimensions, the paper develops a framework for assessing when and how autonomy-sensitive behavioral interventions can be ethically justified in contexts marked by despair and hopelessness.

## Introduction

In 2023, the USA recorded approximately 105,000 drug overdose deaths (CDC, 2024), around 48,000 alcohol-related deaths, and 50,000 suicides (Warren and Reavis, 2025). In total, more than 200,000 Americans died from drug overdose, alcohol misuse, and suicide, twice the number recorded in 1999. Across Europe, similar though uneven patterns have emerged. According to the European Union Drugs Agency (2025), drug-induced deaths remain concentrated among older adults, with a more than 2-fold increase in drug-induced deaths among adults aged 50–64 between 2013 and 2023, reflecting an aging cohort of opioid users. Alcohol-related mortality has also remained a major concern: in the UK, alcohol-specific deaths reached 10,473 in 2023, the highest number ever recorded (Office for National Statistics, 2025). Meanwhile, suicide rates across most of Europe have remained relatively stable or declined slightly over the past decade, though the downward trend has plateaued in recent years (Zuin and de Leo, 2025).

The notion of deaths of despair (DoD) has become a pivotal framework for understanding this troubling trend in contemporary public health. The phenomenon was first documented by Anne Case and Angus Deaton (2015), who identified rising mortality among less-educated Americans driven by suicide, drug overdose and alcohol-related disease. The expression ‘deaths of despair,’ first introduced by Case (2015) and developed in Case and Deaton’s later book (2020), captures the intersection of economic insecurity, social fragmentation and psychological distress underlying these mortality trends. Case and Deaton’s findings revealed a reversal of decades-long declines in mortality among White Americans aged 45–54, particularly those with only a high school education or less. By 2013, this group was seven times more likely to die from drug and alcohol poisoning, five times more likely to succumb to alcohol-related liver disease, and twice as likely to die by suicide as their college-educated peers. According to the authors, these patterns underscore the possible role of economic dislocation, reduced wages and limited social protections in shaping health outcomes.

The urgency of addressing DoD has only intensified as recent evidence documents comparable or higher mortality among other populations (Akee *et al.*, 2024; Friedman and Hansen, 2024; Harris, 2024).

Systemic reforms (such as universal healthcare or legislation protecting workers’ rights) address the structural determinants of these deaths at the level Chater and Loewenstein (2022, 2026) call the *s-frame*. However, where such reforms remain politically or culturally constrained, behavioral public policy (BPP) offers a complementary, actionable *i-frame* (Chater and Loewenstein, 2022, 2026) response that targets despair and hopelessness at the individual level. Drawing on behavioral economics and psychology, BPP develops interventions sensitive to people’s bounded rationality and environmental contexts. The conceptual, empirical, and ethical dimensions of applying BPP to the DoD crisis are explored in the sections that follow.

Philosophical debates about autonomy are central to evaluating such interventions, particularly in the context of DoD, where individuals’ capacity for self-governance may be compromised by emotional, cognitive or structural constraints. Initially, Kant (1785) defined autonomy as the capacity to act according to self-legislated moral principles grounded in reason, independent of external influence. Contemporary approaches, such as Beauchamp and Childress (2019), reinterpret autonomy in practical, relational terms, emphasizing intentionality, understanding, and freedom from controlling influences. This evolution reflects a move from autonomy as purely rational self-legislation to autonomy as situated self-governance, shaped by psychological and social conditions. In medical ethics, autonomy is often seen as either intrinsically or instrumentally valuable. Varelius (2006) argues that within medicine, autonomy should be considered instrumentally valuable, since its moral weight lies in promoting well-being rather than serving as an end in itself.

Heteronomy, by contrast, arises when external forces (manipulation, coercion or unexamined impulses) undermine rational self-governance. Ethically designed BPP interventions, such as nudges, seek to avoid this by guiding individuals toward beneficial outcomes without compromising their reflective agency.

Despite its empirical robustness, the DoD framework faces growing conceptual scrutiny. While midlife mortality increases are well established, scholars question whether ‘despair’ functions as a coherent or measurable construct. Large-scale studies (e.g. Gaydosh *et al.*, 2025) show that despair predicts drug-use behaviors better than it predicts suicidal ideation or heavy drinking, with distinct pathways to each, drawing into question despair’s relevance as a unifying framework. These findings affirm the mortality crisis yet suggest that despair may serve more as a metaphor than a unified causal category. This ambiguity calls for greater conceptual precision. We argue that distinguishing despair from two forms of hopelessness—doxastic and desiderative—provides the most policy-relevant framework for understanding how emotional and cognitive states mediate the link between social dislocation and self-destructive outcomes.

Kwong’s recent work (2019, 2020, 2022, 2024) provides such a framework, distinguishing despair and hopelessness as distinct affective and cognitive states. Despair involves negative emotions toward desired outcomes still perceived as attainable, while hopelessness entails either the loss of desire, or the loss of belief in the possibility of achievement, or both. In its most severe form, when desire itself collapses, hopelessness produces a full breakdown of the preference structure: individuals can neither rank nor pursue goals, resulting in profound disengagement. When only belief in possibility collapses, desires persist but become motivationally inert, unconnected to any sense that action could lead anywhere. These distinctions are crucial for ethics and public policy because despair retains a foundation of agency that can be supported, whereas hopelessness requires rebuilding the emotional and cognitive structures necessary for autonomy.

Integrating Kwong’s distinctions with BPP offers a novel perspective on how interventions can address the emotional roots of self-destructive behavior. Interventions should foster hopefulness in those experiencing despair and reestablish the basic conditions for autonomy among the hopeless, always within ethical boundaries that preserve agency.

In sum, this paper makes a dual contribution. First, it sharpens the conceptual space by distinguishing despair from two forms of hopelessness, desiderative and doxastic. Second, it maps these states onto autonomy-sensitive tools of BPP, including nudges, boosts and sludge audits, with clear ethical guardrails. By integrating philosophical analysis with applied policy reasoning, it explores how BPP can address emotional states that undermine autonomy and agency. The remainder proceeds as follows: the second section develops the conceptual framework of despair and hopelessness; the third section situates BPP as a response to DoD; the fourth section presents specific BPP interventions for each condition; and the fifth section analyzes the ethical implications for autonomy and policy design.

## Conceptual Framework: Despair, Hopelessness, Hoping and Being Hopeful

A nuanced conceptual framework is essential to understand and effectively address DoD. A quick examination may push us to believe that there is no better way to fight despair than a dose of hope. As Esther Duflo argues (2012), hope should be seen not merely as an emotion but as a capability (a precondition for agency that enables individuals to envision and pursue better futures). The erosion of hope thus signals more than emotional decline; it reflects a restriction of one’s freedom to act and to transform opportunities into meaningful functionings. Bovens (1999) deepens this point from a philosophical angle, arguing that hope carries intrinsic value as a psychological orientation that sustains agency, making its loss not merely an emotional misfortune but a normatively significant harm. Together, these perspectives make understanding, cultivating and restoring hope a policy imperative, particularly in contexts where economic and social dislocation erode individuals’ sense of control.

However, hope and despair are like paintings rendered with a thick and broad brush: vivid but imprecise. To grasp their true complexity, we must turn to finer strokes and thinner brushes that can reveal the subtle contours and shades of these experiences. No work has done more to bring such clarity than that of Jack Kwong, who distinguishes despair from hopelessness and their positive counterparts, hoping and being hopeful.

In his work, Kwong identifies two senses of hope: hoping and being hopeful (2020, 2022). Hoping involves desiring an outcome, believing it is possible, and consciously registering that one has the desire; whereas being hopeful reflects a positive emotional and cognitive orientation toward the possibility or chances that the outcome will obtain. This distinction is essential for capturing and understanding how individuals experience and lose hope, as it is possible to hope for something without feeling hopeful. For example, one might say, ‘I hope racism disappears, but I am not hopeful.’

These two senses of hope allow us to identify, with precision, two distinct ways in which hope can be lost. Despair is the loss of what distinguishes being hopeful from merely hoping: the positive orientation toward possibility. A person in despair retains both desire and belief that their goal is achievable but can no longer sustain an optimistic (or otherwise positive) emotional stance toward it. Hopelessness, by contrast, is the loss of the minimal conditions for hoping itself: either the desire collapses, or the belief in the possibility collapses, or both. Focusing on the cognitive dimension alone: in despair, the outcome is still judged possible, but the individual’s attitude toward their chances is negative; in hopelessness, the outcome is no longer judged possible at all. It follows that despair and hopelessness are losses of fundamentally different things, and that hoping alone, in the absence of hopefulness, may not be sufficient to sustain action: a person can hope for an outcome while remaining paralyzed by a negative orientation toward it. This distinction has direct implications for policy interventions, as restoring hope and restoring hopefulness require different strategies.

Kwong’s definition of hopelessness implicitly reveals a subtle yet significant distinction between two forms: desiderative and doxastic hopelessness. Although not explicitly articulated, these forms emerge from his conceptual framework. Desiderative hopelessness is characterized by a complete collapse of desire, leaving individuals without the motivational basis for goal-directed behavior. We acknowledge, following Elster’s reading of the Fox and the Sour Grapes, that the loss of desire does not logically entail the loss of belief: one can genuinely stop caring while still recognizing that a goal is achievable. However, for the purposes of behavioral intervention, a belief in possibility that is no longer attached to any desire becomes functionally inert: it is no longer pursued, entertained, or experienced as actionable. Restoring engagement in desiderative hopelessness therefore requires addressing both the reactivation of desire and the renewal of belief as a motivationally live possibility. In this state, autonomy is profoundly compromised, as individuals lack the capacity to act or make decisions aligned with any values or aspirations. In contrast, doxastic hopelessness occurs when individuals retain desires and preferences but hold a level of credence in the possibility of success that falls below the threshold required for genuinely believing the outcome attainable, creating a paralysis of action despite the presence of underlying aspirations.

For example, consider someone who has stopped applying for jobs entirely, no longer caring to be employed regardless of the opportunities. This collapse of desire exemplifies desiderative hopelessness. Contrast this with someone who still wants to find work but is convinced that no opportunities exist for someone like them: the desire remains, but belief in the possibility of success has collapsed, exemplifying doxastic hopelessness. Despair, by contrast, involves maintaining desires and beliefs in possibility but adopting a negative emotional stance toward achieving them. For instance, a job seeker who continues applying to positions despite constant rejections might feel despair, not because they have stopped hoping, but because they have stopped being hopeful, that is, they struggle to remain emotionally and cognitively positive about their chances. Similarly, a struggling entrepreneur may keep hoping for their business to stabilize but feel overwhelmed by the challenges ahead, making it difficult to maintain a positive outlook on the chances that their hopes will be realized.

All these distinctions can also inform remedies for regaining hope in general. Hopelessness would require interventions that restore desire or belief in possibility, such as demonstrating achievable alternatives or fostering new goals. For instance, public health campaigns showcasing stories of individuals who have overcome addiction can reignite belief in the possibility of recovery. Despair, on the other hand, would demand interventions that cultivate hopefulness by encouraging optimism and clarifying realistic pathways to success. For example, progress-tracking tools in healthcare settings can remind patients of incremental improvements, helping them focus on achievable milestones rather than overwhelming challenges. All these behavioral interventions must be based on realistic and achievable possibilities, so individuals are not tricked into false hope.

While autonomy is constrained in both despair and doxastic hopelessness, it is not entirely absent. In doxastic hopelessness, individuals continue to value certain outcomes, even as they perceive those outcomes as unattainable. In despair, individuals recognize the difficulty of achieving desired outcomes but still retain a degree of agency tied to their aspirations. Accordingly, interventions should rebuild both desires and beliefs in desiderative hopelessness and primarily restore belief (while working with existing preferences) in doxastic hopelessness.

By distinguishing these phenomena, we gain a deeper understanding of how they impact preferences and autonomy, offering valuable insights for addressing issues like Case and Deaton’s DoD. These distinctions lay the groundwork for exploring how BPP tools can address despair and both forms of hopelessness while balancing autonomy and paternalism.

## BPP and DoD

Case and Deaton trace DoD to structural forces (globalization, economic stagnation and the erosion of social safety nets) with education level, race and age as key correlating factors. These systemic causes call primarily for structural remedies: labor market reform, strengthened welfare provision and investment in communities hollowed out by deindustrialization. Yet structural reform faces persistent political and institutional resistance, and in the meantime people die. BPP offers a complementary, not a competing, response. Following Chater and Loewenstein’s (2026) distinction between i-frame interventions targeting individual behavior and s-frame interventions targeting social and institutional structures, we argue that i-frame tools are justified precisely when s-frame change is blocked or delayed, provided they are never permitted to substitute for the structural reforms that remain the primary obligation.

The structural forces they identify produce despair and hopelessness through different pathways, and this difference has direct implications for how BPP intervenes. A laid-off worker who keeps applying for jobs but grows emotionally exhausted exemplifies despair, potentially turning to harmful coping mechanisms such as substance use or self-harm (Barnes *et al.*, 2016; Azagba *et al.*, 2021). Another worker who ceases job-seeking altogether, convinced that no opportunities exist for someone like them, exemplifies hopelessness (action collapses not from emotional exhaustion but under the weight of a perceived impossibility of success). In its most severe desiderative form, where desire itself has collapsed across multiple domains of life rather than being redirected or refined, hopelessness can lead to social withdrawal and preventable death. This distinguishes pathological desiderative hopelessness from the adaptive letting-go of a specific unattainable goal, which may be experienced as liberating rather than debilitating. These differences are not merely descriptive: they reveal distinct ethical and psychological challenges for autonomy and well-being, and they call for distinct intervention strategies.

The structural roots of these states are well documented. Beseran *et al.* (2022), reviewing the US evidence, identify low socioeconomic position, low education, job insecurity, unemployment and rural residence as the most relevant social determinants of these deaths, with declining income and socioeconomic status affecting vulnerable groups. Barnes *et al.* (2016) and Baumann *et al.* (2007) link socioeconomic deprivation to self-harm and substance abuse. These systemic and psychological factors jointly erode agency, leaving individuals unable to act in their best interest. While structural reforms remain the ideal response, BPP offers practical tools that work within existing constraints by applying behavioral insights to promote welfare-enhancing choices without coercion.

BPP emerged to address the limitations of neoclassical economics, which assumes fully rational agents with stable preferences and limitless cognitive capacity. Herbert Simon’s notion of bounded rationality (1947, 1956), followed by Tversky and Kahneman’s (1974) research on cognitive biases, reframed decision-making as context-dependent and shaped by mental constraints. This foundation enabled the development of behavioral policy instruments, most prominently nudges, libertarian paternalistic modifications of choice environments that encourage welfare-enhancing behaviors without coercion (Thaler and Sunstein, 2008). According to their proponents, nudges guide individuals toward better decisions while preserving autonomy and self-determination.

These interventions have been successfully applied to promote healthier eating habits (Velema *et al.*, 2018), reduce gender-based violence (Hortal, 2023), decrease vaccine hesitancy (Hortal, 2022), and curb corruption (Hortal and Pérez Martínez, 2026). For example, Schubert and Schmidt (2024) discuss how nudges such as default options for initiating treatment and appointment reminders may reduce barriers to engaging with mental health care. By aiming to increase access while respecting individual agency, these interventions suggest how behavioral tools might promote well-being without infringing on autonomy.

Their theoretical respect for autonomy, combined with empirical effectiveness and low implementation costs, has contributed to their widespread adoption across fields including economics, health, education and environmental policy. According to Nesterak (2021), ‘More than 600 behavioral science units have been established in governments, businesses, nonprofits and research centers around the world, each drawing inspiration from [Thaler’s] research, with *Nudge* serving as a common language and touchpoint for those aiming to integrate a more realistic view of human behavior into their policies, products, and designs.’ Alongside nudges, the BPP toolkit includes boosts that build decision-making skills (Hertwig and Grüne-Yanoff, 2017), *budges* and *shoves* involving behaviorally informed mandates or prohibitions (Oliver, 2018), and *sludge audits*, which identify and eliminate unnecessary administrative frictions, such as complex forms, long wait times or opaque eligibility criteria, that prevent individuals from accessing services they are entitled to (Sunstein, 2022).

Central to nudge theory is the concept of choice architecture: the intentional design of decision environments to account for cognitive limitations and biases. By structuring choices in ways that facilitate welfare-enhancing decisions, choice architecture offers a powerful mechanism for addressing the psychological and emotional barriers underlying despair and hopelessness. The following paragraphs explore these tools as practical strategies for fostering hope, engagement, and recovery in the fight against DoD.

Proponents such as Sunstein argue that individual behavioral interventions, such as nudges, can complement systemic reforms. In Why Nudge? (2014), he argues that nudges can indirectly influence systemic issues through their cumulative effects. For instance, default enrollment in retirement plans increases individual savings, which can reduce societal reliance on welfare programs. Similarly, energy-saving nudges, such as comparing electricity usage with neighbors, can lead to substantial reductions in carbon emissions when widely adopted. Sunstein highlights how these small behavioral changes, when scaled, can help address broader societal challenges. However, this perspective is not without its critics.

As mentioned, Chater and Loewenstein (2022, 2026) provide a compelling critique of the overreliance on individual-level interventions (i-frame), arguing that framing societal problems at the individual level often yields modest results and diverts attention from systemic, or ‘s-frame,’ solutions that target structural issues. The authors stress how corporate interests sometimes exploit the i-frame to undermine regulatory and systemic reforms. Using examples like climate change, obesity and retirement savings, they emphasize that meaningful policy impact often requires a shift toward systemic-level changes while leveraging behavioral science to support and implement these broader reforms.

This critique underscores the importance of balancing individual and systemic approaches in addressing complex crises like DoD and hopelessness. While systemic solutions often provide the most comprehensive remedy, individual-level interventions remain vital, particularly in contexts where systemic change faces significant political, cultural or logistical barriers, such as universal health care in the United States. The interplay between these approaches highlights fundamental questions about the scope and justification for paternalism in BPP, especially in addressing despair and hopelessness.

## BPP Interventions for Despair and Hopelessness

Building on the conceptual distinctions between despair and hopelessness, this section explores practical applications of BPP. Addressing hopelessness requires interventions that reignite hope by fostering desires and beliefs, either by constructing both anew in desiderative hopelessness or by restoring belief in doxastic hopelessness. For despair, behavioral interventions, in the absence of structural changes, focus on fostering hopefulness, helping individuals emotionally reengage with their desired outcomes. The following examples illustrate how BPP strategies, such as nudges and boosts, can address these psychological states, offering tailored solutions to foster hope and promote meaningful engagement among vulnerable populations. These interventions must always be grounded in realistic material possibilities to ensure they do not inadvertently create false hope, which could further exacerbate disillusionment.

### Interventions for Despair

Despair interventions target the emotional orientation of individuals who retain both desire and belief but can no longer sustain a positive stance toward their goals. The aim is not to change what individuals want or what they believe is possible, but to shift how they feel about their chances when those are realistic.

Cognitive-behavioral interventions can complement nudges here by reframing negative thought patterns, reducing pessimism and fostering a more hopeful mindset toward desired outcomes. Unlike nudges, which work by structuring choice environments, these cognitive-behavioral interventions are better understood as boosts (Hertwig and Grüne-Yanoff, 2017): they build deliberative capacity and equip individuals with skills to manage their own thought patterns, rather than steering behavior through environmental design. The behavioral economics literature offers extensive insights into optimism bias and pessimism, highlighting their influence on decision-making. Studies have explored how unrealistic optimism or pessimism shapes the decisions individuals make under uncertainty (Costa-Font *et al.*, 2009; Sharot, 2011; Bracha and Brown, 2012; Smith, 2015).

For example, Dickinson and Oxoby (2011) demonstrated how pessimism can spill over from one context into other areas of life, negatively impacting behaviors such as economic decision-making. Persistent pessimism can reduce engagement and effort across various aspects of life, perpetuating a cycle of inaction. As the authors put it, their ‘experimental methodology highlights how events can ascribe beliefs, for good or bad, which implies that one’s optimism or pessimism is built and rebuilt on a constant basis’ (Dickinson and Oxoby, 2011: 302–303). This finding suggests that optimism or pessimism is not fixed but instead continuously influenced by experiences and circumstances. Even individuals who are dispositionally pessimistic can have their outlook temporarily shifted toward optimism when they encounter reinforcing positive events or experiences. Altering choice environments through nudges and other BPP tools can harness these dynamics, creating spillover effects where an induced sense of optimism in one context extends to other areas of life, fostering more positive behaviors and decisions. However, it is crucial to carefully consider the material conditions and available resources to ensure that interventions are grounded in achievable outcomes, avoiding the risk of fostering false hope.

Framing nudges are particularly effective here. Presenting recovery statistics positively (for example, ‘8 in 10 people who complete this program remain after one year’ rather than ‘20 per cent relapse’) does not provide new information about achievability (that is the target of doxastic hopelessness interventions) but reframes existing information to support a more optimistic emotional orientation. The individual already believes recovery is possible; the nudge helps them feel differently about it.

Progress tracker applications such as Sober Grid (an app that provided peer support and resources for individuals in recovery from substance use) or NOMO (a sobriety clock app) worked similarly. By sending milestone notifications (‘You have been sober for 90 days’ or ‘You have attended 12 sessions’) they provide periodic emotional reinforcement of achievements the individual is already pursuing. It is important to note that the same tool can address doxastic hopelessness through a different mechanism: when a person believes recovery is impossible, a progress tracker provides epistemic evidence that improvement is occurring. For a person in despair, who already believes improvement is possible, the same tracker instead shifts emotional engagement by making progress visible and felt. The distinction lies not in the tool but in what the tool targets.

Social-norm nudges operate similarly. Telling a patient that ‘most people in your situation who sought help reported improvement within 8 weeks’ can function as a despair intervention by modeling the emotional stance of others, showing that people in comparable circumstances maintained optimism and were vindicated. This differs from its use in doxastic hopelessness, where the same message functions epistemically, providing evidence that the outcome is achievable rather than modeling how to feel about it.

### Interventions for Doxastic Hopelessness

Doxastic hopelessness interventions target individuals who retain desires and preferences but have lost belief in the possibility of achieving their goals. The aim is epistemic: to provide concrete evidence that the outcomes they still desire are in fact attainable.

Community nudges work primarily through this epistemic mechanism. Automatic enrollment in peer support networks or invitations to community events introduce individuals to others who have successfully navigated similar hardships. This provides vivid, concrete evidence that outcomes previously deemed impossible are in fact achievable, directly targeting the collapsed belief. The nudge restores hope by demonstrating that success is possible, not by changing how the individual feels about their goals, but by changing what they believe about their chances.

Progress tracker applications offer a related mechanism. For a person in doxastic hopelessness, who wants to recover but believes it is impossible, each completed milestone constitutes direct evidence against the belief that success is unachievable. This differs from their use in despair: for a despairing person who already believes recovery is possible, the same tracker functions as emotional reinforcement rather than epistemic correction. The distinction lies not in the tool but in what the tool targets.

Social-norm nudges also function epistemically here. Telling a patient that ‘most people in your situation who sought help reported improvement within 8 weeks’ provides evidence that the outcome is achievable rather than modeling how to feel about it, which is the mechanism in despair interventions.

In the context of chronic pain, belief-reinforcement nudges can challenge the assumption that improvement is unattainable. For instance, digital health apps might share success stories of individuals who, through consistent treatment, have significantly reduced their pain levels. Personalized reminders tailored to an individual’s pain profile could emphasize small but meaningful progress, reinforcing the belief that improvement is achievable. Progress trackers, often more accepted when run by AI or algorithms (Raveendhran and Fast, 2021), can visually display incremental improvements in mobility or pain management, fostering hope through measurable achievements. Additionally, nudging individuals toward peer support groups for chronic pain sufferers can rebuild belief in positive outcomes by providing examples of others who have managed their conditions successfully.

These nudges address the core cognitive barriers of doxastic hopelessness by demonstrating that improvement is possible and desirable. They encourage belief in the potential for success, even if emotional optimism has not yet fully returned.

Goal-setting applications that break a large goal (sustained employment, addiction recovery, financial stability) into small daily milestones make incremental progress visible and measurable. For a person in doxastic hopelessness, each completed step represents direct proof against the belief that success is unachievable. This is different for a despairing person, who already believes recovery is possible, in which case the same app functions as emotional reinforcement rather than epistemic correction. Auto-enrollment nudges, such as default registration in job-training programs or addiction-recovery groups, work through a related mechanism: by lowering the threshold for engagement, they give individuals the experience of participation, which itself can begin to shift the perceived probability of success.

Sludge audits are particularly effective in doxastic hopelessness by eliminating bureaucratic barriers to accessing essential resources, such as job training or mental health services. For individuals who already desire change but believe it is impossible, reducing administrative friction lowers the cost of a first attempt, which can itself begin to shift the perceived probability of success. Simplifying enrollment in addiction-recovery or welfare programs restores a sense of efficacy by demonstrating that the system is navigable.

### Interventions for Desiderative Hopelessness

Desiderative hopelessness presents the most demanding intervention challenge because there are no existing desires to work with. The aim is not epistemic (restoring belief in possibility) nor attitudinal (shifting emotional orientation toward existing goals), but motivational: introducing new objects of care that can reactivate the preference structure from the ground up. This is also the most ethically sensitive application, as it involves introducing desires from outside the individual’s existing preference set, a point we return to in the fifth section.

Social connectedness nudges are among the most versatile BPP tools here. Activities such as volunteering, joining a community group or participating in shared projects introduce new objects of care, things worth doing and looking forward to. The nudge is not providing evidence of achievability nor shifting emotional orientation toward existing goals, but offering new goals to desire in the first place.

In cases where social isolation cannot be resolved, other nudges can focus on increasing well-being directly. For example, the Happiness Route intervention (Weiss *et al.*, 2013) targets individuals facing social isolation, health issues or low socioeconomic status, factors closely linked to desiderative hopelessness. This approach encourages people to set positive, achievable goals and engage in intrinsically rewarding activities that enhance emotional, social and psychological well-being. Such nudges align with hope-focused interventions by fostering new desires and reinforcing beliefs in attainable outcomes.

Behavioral Activation, a structured behavioral treatment for depression (Martell *et al.*, 2010), offers a more systematic approach to the same problem. Although boosts are typically framed as competence-fostering interventions (Hertwig and Grüne-Yanoff, 2017), Behavioral Activation can be read as boost-like in a broader sense: rather than steering a choice, it rebuilds the motivational substrate from which the person’s own reasons to act can reemerge. This is also where it departs from a typical boost, since the mechanism is behavioral rather than cognitive: the goal is not to change how the person thinks about their situation but to reactivate engagement through doing. For a person in desiderative hopelessness, the program does not work by showing that existing goals are achievable, since there are no existing goals to target, but by introducing new activities that generate fresh reasons to care. This distinguishes it clearly from its use in despair, where Behavioral Activation would instead reengage the person with activities connected to goals they already hold, and from doxastic hopelessness, where the person already knows what they want and needs evidence that it is achievable rather than new sources of motivation.

Structured volunteering schemes illustrate the same logic at the community level. Programs such as AmeriCorps in the USA or the UK’s National Citizen Service introduce participants to new communities, new competencies and new identities. The mechanism is motivational: the individual is not being shown that their goals are achievable, nor having their emotional orientation shifted toward existing goals, but being offered entirely new objects of care. The ethical sensitivity of this approach, introducing desires from outside the collapsed preference set, is addressed in the following section.

### A Note on Crossover

Many of the tools discussed across these three sections, including progress trackers, community nudges, social-norm nudges and sludge audits, appear in more than one subsection. This is not a conceptual inconsistency but a reflection of the fact that the same tool can address different psychological states through different mechanisms. A progress tracker provides emotional reinforcement in despair, epistemic correction in doxastic hopelessness, and, when linked to new activities, motivational scaffolding in desiderative hopelessness. Real cases will often blend elements of all three states, and interventions should be designed with this in mind. The three-way distinction is analytical: it identifies the primary mechanism each intervention targets, not a claim that individuals fall neatly into one category.

The personalization of interventions should be a cornerstone of BPP, enhancing both efficacy and efficiency (Ruggeri *et al.*, 2023). Despair, marked by emotional barriers to achieving desired outcomes, requires interventions that reinforce optimism and engagement, while hopelessness, whether desiderative or doxastic, necessitates strategies that rekindle desires, beliefs, or both. By understanding individuals’ bounded rationality and cognitive limitations, BPP provides targeted support that aligns with their unique needs and circumstances. The next section examines the ethical considerations that govern the design and justification of these interventions.

## Balancing Autonomy and Paternalism in BPP: Ethical Reflections on Despair and Hopelessness

### The Ethical Foundations of BPP

The ethical legitimacy of BPP interventions hinges on how they balance welfare promotion with respect for individual autonomy. This balance becomes especially delicate when policies address vulnerable psychological conditions such as despair and hopelessness, where autonomy itself is impaired or eroded. Thaler and Sunstein (2003, 2008) introduced libertarian paternalism as a framework that reconciles welfare promotion with individual freedom, arguing that paternalistic interventions can be justified if they preserve the capacity for choice.

BPP draws on behavioral economics to design interventions, such as nudges, that subtly influence decision-making in ways that promote well-being without coercion. These interventions rely on choice architecture: carefully structured decision environments that mitigate cognitive biases and self-control failures while preserving freedom of choice. Rather than removing or restricting options, nudges modify how choices are presented, enabling individuals to make decisions they are likely to judge as beneficial in hindsight. Sunstein (2020) argues that nudges empower individuals to act in their long-term interests by addressing bounded rationality and self-control issues without resorting to coercion. Hansen (2016) further refines this view, showing that nudges can maintain autonomy by correcting biases while ensuring individuals retain ultimate control over their decisions.

The framework of nudges incorporates a nuanced and multifaceted conception of autonomy. While earlier sections traced autonomy from Kant’s classical account of rational self-legislation through to contemporary relational and practical conceptions, contemporary scholars such as Vugts *et al.* (2020) expand on this concept by identifying three interrelated components: freedom of choice (availability of options), agency (capacity for deliberation) and self-constitution (alignment of choices with one’s personal identity and goals). Proponents of nudges claim that these interventions respect and support all three dimensions by structuring environments that facilitate informed and welfare-enhancing outcomes. Krpan and Urbaník (2024) add that technologies such as gamification and self-quantification could enhance this libertarian dimension, allowing individuals to personalize behavioral interventions in line with their own goals. Yet this multidimensional view of autonomy remains contested, especially when applied to real-world contexts where the tension between guidance and freedom becomes ethically salient.

Despite these claims, critics argue that nudges are not as libertarian as their advocates suggest. Gigerenzer (2015), for example, contends that behavioral economics relies on an overly simplistic view of human irrationality and suffers from a ‘bias bias’ (2018). He argues that this perspective undermines autonomy by failing to equip individuals to develop decision-making competence. Instead of steering behavior through choice architecture, Gigerenzer promotes risk literacy, empowering individuals with the knowledge and skills to make independent, informed decisions (2014).

Other scholars extend this critique to the paternalistic assumptions embedded in nudges. Rizzo and Whitman (2019) argue that nudges infantilize citizens by assuming policymakers possess superior knowledge of welfare, a concern first raised in the BPP literature by Bovens (2009) and developed influentially by Waldron (2014). Grüne-Yanoff (2012) claims that they impose normative assumptions about what is ‘better’ for individuals, undermining genuine autonomy. McCrudden and King (2016) highlight the political risks of nudging, arguing that behavioral influence can operate covertly and embeds, often insidiously, within a broader libertarian program of political economy. These critiques converge on the idea that, while nudges avoid overt coercion, they nevertheless steer individuals toward predefined outcomes, revealing an inescapable paternalistic core. The central question, therefore, is not whether nudges are paternalistic, but how they might ethically balance paternalism with respect for autonomy.

### Ethical Implications of Tailored Interventions

The personalization of BPP interventions ensures they effectively address the unique psychological states of despair and hopelessness. By tailoring strategies to individuals’ needs and emotional barriers, BPP empowers people to move toward recovery and a renewed sense of purpose, bridging the gap between emotional distress and actionable hope. However, these interventions raise significant ethical considerations, particularly regarding autonomy and consent. While less coercive than traditional policies, they still guide individuals toward specific outcomes, which can blur the line between influence and manipulation.

Scholars like Hill (2018) argue that nudges, though subtler than mandates, can create ethical tensions by subtly steering behavior while preserving the appearance of choice. This concern becomes particularly salient when interventions target foundational hopelessness, where individuals lack both the desires and beliefs needed to form autonomous decisions. In such cases, greater paternalism (with greater ethical risk) may be required to reignite desires and foster belief in achievable outcomes, but this risks imposing external values or preferences. Conversely, interventions for doxastic hopelessness, where desires persist but belief in their achievability is absent, align more closely with existing preferences, making them less ethically fraught.

Interventions for despair, where desires and beliefs remain intact but emotional engagement is lacking, face a different ethical balance. Strategies to foster hopefulness can enhance autonomy by supporting individuals in reconnecting with their goals. Yet even here, policymakers must navigate the tension between empowerment and overreach, ensuring transparency in the design and implementation of interventions.

The risk of external desire imposition in desiderative hopelessness is not merely theoretical, and it is worth dwelling on cases that should give policymakers genuine pause. Consider a faith-based addiction-recovery program that conditions access to counseling, housing support and peer networks on participation in religious observance. For a person in desiderative hopelessness, the program does not merely support recovery; it colonizes the preference set that is being rebuilt. The desires that reemerge may be the program’s desires rather than the individual’s own. The intervention has been effective in a narrow clinical sense while violating autonomy in a deeper one.

A second case: commercial wellness platforms that nudge unemployed or socially isolated individuals toward productivity goals, consumption habits, or lifestyle aspirations aligned with the platform’s commercial interests. Here the concern is not that the desires introduced are harmful, but that they are exogenous, imported from a commercial interest rather than emerging from the individual’s own history, values or reflective preferences. When desire has collapsed entirely, the person cannot distinguish between a desire that is genuinely their own and one that has been installed by an intervention.

A third case: government employment programs that channel individuals experiencing desiderative hopelessness toward sectors with labor shortages, framing participation as ‘finding purpose’ or ‘rediscovering motivation.’ The structural interest of the state in filling particular roles may not align with the individual’s emergent authentic preferences, yet the person in desiderative hopelessness has no existing preference structure from which to resist or evaluate the channeling. These cases collectively illustrate why interventions for desiderative hopelessness require a higher standard of ethical scrutiny than those for despair or doxastic hopelessness: the absence of desire is not simply a problem to be solved, but a condition of profound vulnerability to normative capture.

Bovens (2021) captures this risk precisely, arguing that coping (the active adjustment of one’s desires and expectations in response to adverse circumstances) is itself a form of agency that interventions should support rather than preempt. The risk in desiderative hopelessness is not merely that external desires are introduced, but that the individual’s own nascent coping processes are bypassed before they have had the chance to generate authentic new preferences.

Cultural sensitivity further complicates the ethical landscape. Interventions that fail to consider diverse cultural norms and social contexts risk imposing external frameworks that conflict with individuals’ lived experiences. Adaptations to align strategies with local values and expectations not only enhance effectiveness but also safeguard autonomy and agency.

### Autonomy, Paternalism and Psychological Vulnerability

Building on the earlier distinctions between despair and the two forms of hopelessness, the enduring tension between libertarian and paternalistic interpretations of nudges provides a normative framework for evaluating their ethical scope. In cases of despair (where individuals retain desires and beliefs but struggle with emotional engagement) nudges can strengthen autonomy by aligning with existing value frameworks and helping individuals reconnect with their goals. By contrast, desiderative hopelessness, characterized by the absence of both desires and beliefs, requires interventions that rebuild autonomy from the ground up. Such measures raise significant ethical concerns, as they risk imposing external values to generate new preferences and beliefs. Doxastic hopelessness, where desires persist but belief in achievability has collapsed, presents a different ethical profile. Here, nudges may focus on restoring belief in possibilities, working within individuals’ existing evaluative frameworks while avoiding the risks of normative overreach associated with desiderative hopelessness.

Gigerenzer’s emphasis on education and empowerment resonates with these challenges, particularly regarding desiderative hopelessness, where paternalistic interventions risk undermining agency altogether. By contrast, despair and doxastic hopelessness allow for less intrusive measures that respect autonomy while supporting self-directed action. Balancing these approaches is essential to designing interventions that remain both ethically defensible and practically effective.

While the literature on DoD provides valuable insights into structural determinants (such as economic dislocation, educational inequality and the erosion of community) it rarely distinguishes between despair and hopelessness. Gutin *et al.* (2023) describe despair as a multidimensional construct linked to suicidality and substance use, but subsume cognitive states such as hopelessness within despair rather than treating hopelessness as a distinct condition marked by the absence of both desire and belief. Similarly, Pecchenino (2015) and Na *et al.* (2022) examine despair as a socioeconomic phenomenon yet fail to address how these states uniquely affect autonomy or how interventions might restore it. This conflation obscures the ethical distinction between empowering interventions (appropriate for despair) and reconstructive ones (necessary but risk-laden in hopelessness).

The justification for paternalistic interventions therefore varies across these conditions. In despair, where beliefs and desires remain intact, nudges and boosts can ethically enhance autonomy by reducing emotional barriers and fostering reengagement with life goals. Hopelessness, by contrast, generally demands more intrusive measures aimed at reconstructing the preconditions for autonomy, though the degree of intrusiveness tracks the form: doxastic hopelessness, where desires persist and only belief in possibility has collapsed, allows interventions that work within an existing preference structure, while desiderative hopelessness, where desire itself has collapsed, poses the sharpest autonomy challenge and requires the most ethically careful reconstructive approach. Such interventions carry higher coercive risks and are justified primarily when addressing systemic barriers or creating minimal conditions for hope. Because large-scale structural reforms are often politically or logistically constrained, BPP offers pragmatic tools for gradually reintroducing a sense of possibility without overtly imposing external values.

### Implications for Policy Design

Understanding how despair and hopelessness differently affect autonomy has direct implications for designing BPP interventions. In despair, policies can enhance autonomy by aligning with individuals’ existing frameworks of desire and belief. Framing nudges (e.g. job-training programs that present success stories) can help individuals recognize viable opportunities and make informed choices, strengthening their perceived control. Social-norm nudges, such as highlighting recovery rates in addiction treatment, can reinforce optimism and self-efficacy, aligning behavior with latent aspirations.

Boosts, such as financial-literacy training or personalized goal-setting programs, further support agency by cultivating decision-making competence. Meanwhile, sludge audits can remove unnecessary bureaucratic barriers that limit access to critical resources. Simplifying enrollment in addiction-recovery or welfare programs, for instance, lowers entry barriers and restores a sense of efficacy. By eliminating these ‘sludge’ elements, individuals facing despair are better able to pursue opportunities that align with their values, supporting their journey toward autonomy and well-being.

In contexts of hopelessness, where autonomy is profoundly compromised, more structural approaches are often required. Measures such as universal basic income can reduce economic precarity and restore basic agency. Complementary behavioral tools can reinforce these efforts, simplifying access to mental health services through sludge audits, or using indirect nudges like community campaigns and public-space design to reintroduce desire and belief in possibility. While such strategies may not immediately restore autonomy, they establish the psychological and material groundwork for its recovery.

Across all contexts, interventions must navigate the ethical boundary between manipulation and empowerment. Nudges and boosts should be designed transparently, preserving the individual’s capacity for deliberation and informed choice. Cultural sensitivity is also vital: behavioral strategies must adapt to local norms and values to avoid imposing external frameworks of meaning. Contextually tailored interventions not only improve effectiveness but also ensure that the process of fostering autonomy remains ethically sound.

Ultimately, the justification for paternalism in BPP depends on the condition it seeks to address. While the ethical rationale for intervening in hopelessness may be weaker (given the depth of compromised autonomy) the moral imperative to alleviate extreme suffering remains strong. Interventions must therefore be designed with exceptional care, prioritizing conditions that allow autonomy to reemerge rather than prescribing externally defined solutions. In this way, BPP can serve not merely as a behavioral technology but also as an ethically informed practice that restores the capacity for self-determination in contexts of despair and hopelessness.

## Conclusion

This article advanced a nuanced understanding of despair and hopelessness by integrating Jack Kwong’s philosophical framework to elucidate their implications for autonomy and BPP in the context of the public health crisis of DoD. It identified two distinct forms of hopelessness within this framework: desiderative hopelessness, defined primarily by the collapse of desire and doxastic hopelessness, where desires persist but the individual’s credence in the possibility of success falls below the threshold required for genuinely believing the outcome attainable. These distinctions are not merely conceptual refinements but provide essential guidance for designing interventions that respect and restore autonomy.

BPP emerges as particularly well-suited to address DoD where large-scale structural reforms (universal healthcare, expanded social safety nets, labor market reregulation) are politically or logistically blocked. While such reforms remain the more direct and effective response to the structural roots of DoD, behavioral interventions can complement these efforts through nudges, boosts and sludge audits, provided they do not become a substitute for the structural changes that remain the primary obligation. Properly designed, such tools can help individuals in despair regain agency by fostering realistic hopefulness, assist those in doxastic hopelessness by rebuilding belief in achievable outcomes, and reintroduce objects of care for those in desiderative hopelessness where desire itself has collapsed. Throughout, interventions must remain grounded in genuinely achievable possibilities: behavioral strategies that foster hope disconnected from real material conditions risk compounding the disillusionment they are meant to address.

Autonomy plays a dual role in this analysis. Conceptually, it is deeply affected by the transitions between despair, doxastic hopelessness and desiderative hopelessness. Ethically, it serves as the criterion for evaluating when paternalistic behavioral interventions can be justified. Autonomy is most compromised in desiderative hopelessness, where rebuilding agency requires exceptional ethical care to avoid the paternalistic imposition of external desires that may not reflect the individual’s authentic values or emergent preferences. In doxastic hopelessness and despair, by contrast, interventions can ethically align with existing values and beliefs, supporting self-directed recovery.

By integrating philosophical distinctions with practical policy design, this article contributes to the growing dialogue between behavioral public health policy and normative ethics. It underscores the need for culturally sensitive, autonomy-centered interventions that are both effective and ethically defensible in addressing one of the most urgent public health challenges of our time. Future research should examine the long-term effects of BPP on autonomy and well-being, and strategies to mitigate the risks of false hope and the limits of behavioral tools in confronting systemic inequities.

Ultimately, BPP’s promise lies in its ability to complement rather than replace structural change. When ethically grounded, it can help foster hope, restore agency and empower individuals confronting despair and hopelessness—reaffirming the role of autonomy not only as a philosophical ideal but also as a practical condition for human flourishing.
